# Exposure of the US population to extreme precipitation risk has increased due to climate change

**DOI:** 10.1038/s41598-023-48969-7

**Published:** 2023-12-08

**Authors:** Jungho Kim, Jeremy Porter, Edward J. Kearns

**Affiliations:** First Street Foundation, Brooklyn, NY USA

**Keywords:** Hydrology, Natural hazards, Civil engineering

## Abstract

The magnitude and frequency of extreme precipitation events in the early twenty-first century have already proven to be increasing at a rate more quickly than previously anticipated. Currently, the biggest consequence of the change in extreme precipitation is the lack of a climate-adjusted national standard taking into account these recent increases that could be used to prevent life and property loss from catastrophic precipitation-driven floods. Here, we address how severe the change in extreme precipitation compares against the current national standard for precipitation climatology (NOAA Atlas 14) and how much of the population is affected by the underestimation of this risk in the contiguous United States (CONUS). As a result, extreme precipitation in the early twenty-first century has outpaced our current national standard in half of CONUS, and the heavy precipitation events experienced recently are quickly becoming a “new normal”, which will increase in severity and frequency in a continually changing climate. Over three-quarters of the U.S. population will likely experience this new normal occurrence of extreme precipitation. As much as one-third of the population is expected to experience the current definition of a 1-in-100-year storm as often as three times in their lifetime. Additionally, the current precipitation standards for designing transportation infrastructure and urban stormwater drainage systems that are built upon Atlas 14 may be insufficient to protect the public's safety and personal/community property from severe flooding. Areas where flood risk is mitigated by operating hydraulic and adaptation structures urgently need to assess the impact of the increased-hourly extreme precipitation and reevaluate their applicable operation rules. Understanding and predicting patterns and the likelihood of short-duration heavy precipitation would be beneficial in preparing for severe precipitation-driven disasters, such as flash floods and landslides, which would happen more frequently in a changing climate. Following the results of this analysis, accelerating the development and dissemination of the next generation of the national standard that has been climatically adjusted to adapt to the new normal is strongly recommended.

## Introduction

Levels of extreme precipitation severity and frequency have increased in recent years due to the observably changing climate. These increases have resulted in catastrophic floods, the likes of which we have not observed in the historical record^1^. Within a 2-week period in 2022, there were five 1-in-1000-year events (July 26th in St. Louis, MO, July 28th in Eastern KY, August 1st in Southeastern, IL, August 5th in Death Valley, CA, and August 22nd in Dallas, TX) illustrating the degree to which extreme precipitation increases are becoming more frequent and extreme this century compared to the previous one. Most notably, precipitation events with a low likelihood (e.g., 1% chance of occurring in any given year for a 1-in-100-year event) are not as rare in the current climate as their historically derived probabilities indicate they should be. As a result, we are likely to experience more frequent flooding in our lifetime than expected, and infrastructure designed based on the current national standards is not able to protect the public from the severity of flooding that could occur in these events. Understanding the current underestimation of the frequency and severity of extreme precipitation events is paramount to align society to the new climate normal while preparing us for potential-catastrophic events like “Arkstorm”, a hypothetical mega-storm scenario causing up to $725 billion in losses and requiring the evacuation of 1.5 million people^2^. To adapt to this new normal, we must understand and quantify the severity of extreme precipitation’s magnitude and frequency changes, while identifying the areas most likely to be underestimating their risk today and into the future. Ultimately, this work will allow for the subsequent updating of both the methods used and the national standards of precipitation frequency estimates (PFE).

Currently, the national standard for our understanding of extreme precipitation risk is provided by the federal government through the National Oceanic and Atmospheric Administration (NOAA) Atlas 14. NOAA Atlas 14 is well-known as the national standard climatology of precipitation frequency and severity has been utilized to assess inland flood risks and set design standards for engineers (and a wide range of other professionals) in the development of infrastructure and construction that could be affected by precipitation driven flooding^3–7^. Atlas 14 has provided precipitation depths for various durations and annual recurrence intervals (i.e., return periods). The vast amount of information in the Atlas 14 precipitation records allows for a wide range of applications in order to understand the consequences associated with many different frequency and duration scenarios (e.g., 1-in-100-year precipitation depth for an hour duration). However, in a warming climate, the atmosphere’s capacity to hold water vapor increases linearly with increasing temperatures, resulting in increased severity and likelihood associated with specific rainfall rates, which are not directly accounted for in the current risk estimates from Atlas 14.

Consequently, the Atlas 14 precipitation records are losing their representativeness of current conditions almost exclusively due to the non-stationarity of extreme precipitation^8^. Indeed, the non-stationarity of meteorological phenomena over time has been accelerated by global warming, and extreme weather events’ severity and frequency have increased faster than expected^9,10^. The expectation of annual maximum precipitation for the recent 20 years, a baseline of precipitation frequency estimates for rare cases in extreme value analysis, has increased as compared to the 20th-century records^11^. Still, we specifically need to quantify how much the “new normal” differs from the previous century’s climatological standard and need to explore what those deviations imply. Thus, investigating the severity of increased extreme precipitation is worthwhile for understanding the new normal in the United States and supporting the water science and engineering communities.

In the middle-late twentieth century, increases in heavy precipitation were mainly investigated on daily timescales due to the limited availability of hourly data^12–14^. Due to temporal variability of precipitation, intensities in an independent storm event are inconsistent by time (e.g., Huff’s distribution)^40^. Intense precipitation generally lasts only a few hours (as opposed to longer periods). Thus, the importance of understanding changes in hourly precipitation cannot be neglected. According to recent studies, the changes in extreme precipitation properties are more notable and significant at hourly durations than at daily durations, spatially and quantitatively^11,15^. Globally, hourly extreme events that occurred once per year in 1979–1988 increased in frequency by 71% in 1989–2018, while the frequency of the daily extreme events per year increased by 44%^16^. The mean frequency increases are three times greater for hourly precipitation over land compared with over the ocean, and two times greater for daily precipitation, suggesting the increase in hourly extreme precipitation’s magnitude and frequency is more severe than that in daily durations^16^.

The increases in surface and dew-point temperatures are the primary reasons we are seeing this growth in frequencies since they lead to increases in the atmospheric moisture holding capacity and the likelihood of heavy precipitation occurrence accelerated by global warming^17–20^. In fact, an increase in hourly extreme precipitation is strongly correlated with surface temperature, and the relationship between them is linear with a regression slope higher than 7%, the approximate Clausius–Clapyron (C–C) rate^18^. Hourly precipitation extremes exhibit enhanced scaling with a growth rate that is equivalent to double the C–C rate^21^. In the northern United States (mostly in the Central and East) there are higher regression slopes, with a median regression slope of 16%, being confirmed^12^. Unfortunately, these facts will accompany other negative cascading effects, such as flash and groundwater flooding, and associated environmental issues, such as inducing surface contaminants into groundwater resources^22^.

Most literature agrees that extreme precipitation in the twenty-first century differs from the last century, but there is disagreement as to how different the frequency and severity are^23–27^. However, it is clear that there are spatial variations in both the frequency and magnitude changes based on a region's climate and the region’s unique topographic features. This is because extreme weather sources (e.g., tropical cyclones or convective storms) and the occurrence probability of extreme precipitation by orographic effects differ depending on the two factors^28^. Northern California and Carolina states, populated areas along the west and east coastlines, as well as the upward slopes of mountain ranges, in the United States are specifically seeing considerable increases in extreme precipitation for sub-daily durations^8,11^. Texas and the eastern states are additionally seeing significant increases in extreme precipitation resulting from tropical cyclones and convective storm systems^13,14,29^. These findings are enough to project a trend of extreme precipitation but not enough to more accurately quantify the level of the changed severity, which could be useful for more practical purposes^8,11,13,14,23–27,29^.

This paper mainly aims to answer how extreme precipitation has severely changed against the national standard and how many populations are affected by this new normal in the contiguous United States (CONUS). The questions are answered by comparing two precipitation frequency standard datasets representing the twentieth and twenty-first centuries. In doing so, this paper directly quantifies how severe the change, mainly in hourly 1-in-100-year events, is compared to the national standard and how much of the population is affected by the new normal across CONUS. This study builds on previous research which developed the new gridded precipitation frequency estimates from the 21st-century records^11^.

## Materials and methods

### Study area

In this study, a change in the severity of increased extreme precipitation is investigated across CONUS. CONUS has diversities in climate zones, geomorphological features, and water vapor sources from the Pacific Ocean, the Gulf of Mexico, and the Atlantic Ocean^11^. Because of this diversity, increases in extreme precipitation and pluvial flooding accelerated by climate change have been observed in many different regions for the last two decades^24,30^.

### National standard: NOAA Atlas 14

NOAA Atlas 14 data used in this study is publicly available from the Precipitation Frequency Data Server of Hydrometeorological Design Studies Center (https://hdsc.nws.noaa.gov/hdsc/pfds/pfds_gis.html). NOAA provides a range of Atlas 14 PFEs representing frequent and extreme precipitation scenarios in the United States. The PFEs for durations from 5 min to 60-day and annual exceedance probabilities from 1/2 to 1/1000, corresponding to 2- to 1000-year annual recurrence intervals, are provided. PFEs were developed based on the regional frequency analysis for all regions in the CONUS and created at 0.00833-degree resolution. This study uses the gridded precipitation frequency estimates and upper and lower confidence limits for a 1-in-100-year average recurrence interval for a 60-min duration at the same spatial resolution.

### Twenty-first-century precipitation frequency data

A new precipitation frequency dataset to represent the twenty-first century was developed by First Street Foundation. The data were developed by accounting for only the most recent 20 years changes in precipitation records. Observations from 794 NOAA Automated Surface Observing Station (ASOS) sites were utilized as the primary precipitation data source and to develop point-based precipitation frequency estimates for 1–24 h durations. Gridded Oregon State University PRISM daily precipitation estimates were used to create a predictor map, and ultimately develop maps of precipitation frequency estimates. The early 21st-century period 2000–2021 was considered in the development of a new precipitation frequency dataset. For more details, readers should refer to the development papers^8,11^.

### Census data

2018 US Census Bureau’s American Community Survey data at a county scale is utilized in the population analysis. The data are publicly available from the United States Census Bureau (https://www.census.gov/en.html).

### Categorization method

**Figure 1:**
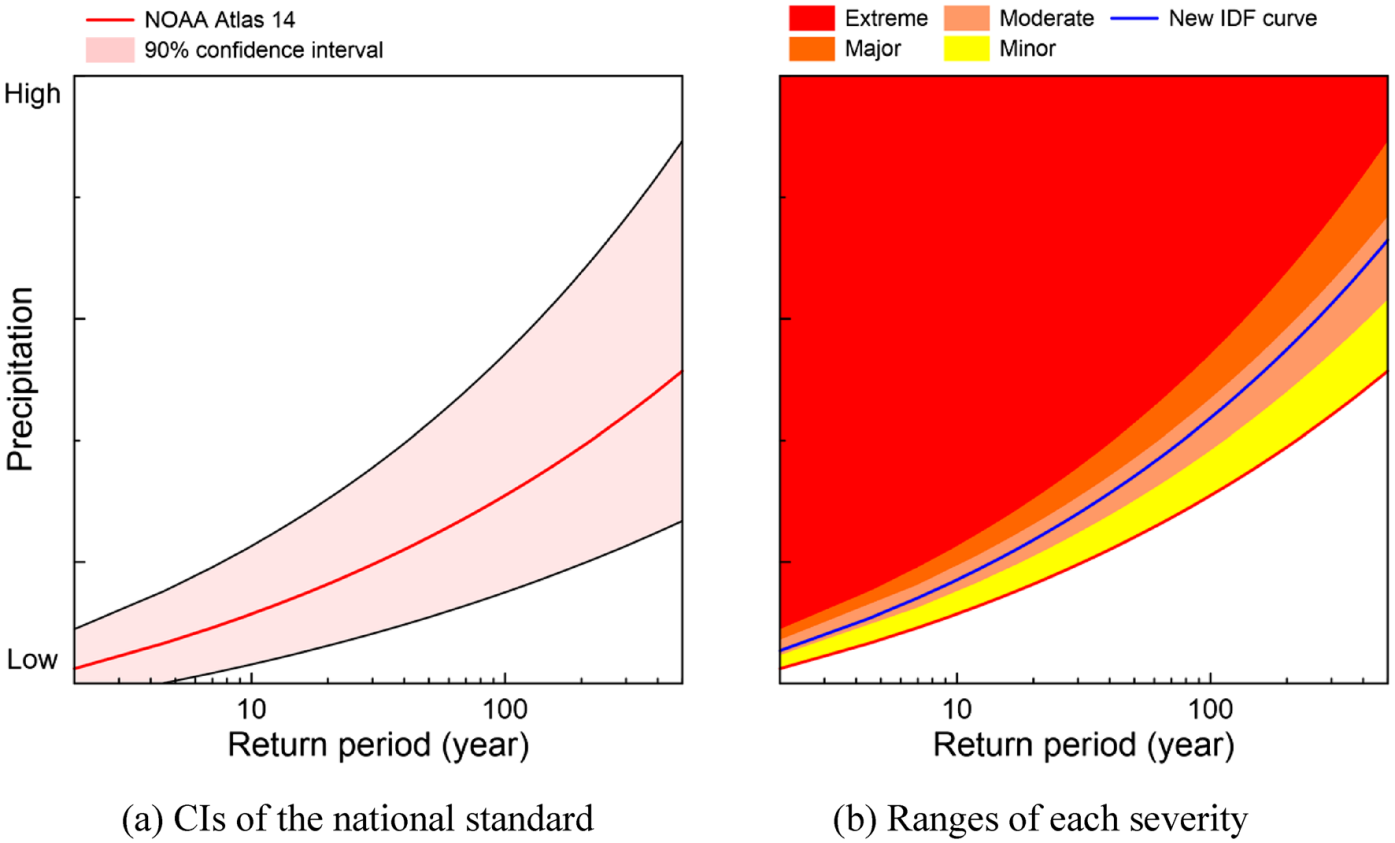
A concept diagram of categorizing severities of changes in extreme precipitation: (**a**) the confidence intervals (CI) of the NOAA Atlas 14, the upper, median (red line), and lower bounds, and (**b**) a range of severities categorized by four categories, minor, moderate, major, and extreme. In the panel (**b**), the blue line demonstrates an example of an intensity-frequency curve for an hour duration (i.e., precipitation frequency estimates) categorized to moderate severity.

A change in extreme precipitation is categorized by accounting for the national standard's confidence intervals (CI). The NOAA Atlas 14 provides 90% CIs as the upper and lower bounds of precipitation frequency estimate, as shown in Fig. 1a. The median and the upper 90% CI of the Atlas 14 are considered as criteria to classify the severity of the increased extreme precipitation in the twenty-first century. A total of four severity levels are defined. The upper 90% CI range defines the first three severities, Minor, Moderate, and Major, beyond the median value but within 90% CI. These severities present a level of increased extreme precipitation against the national standard. They are classified by equally dividing the range into exactly three portions, as shown in Fig. 1b. Extreme severity indicates a case of increased extreme precipitation over the upper bound of the national standard, meaning that the national standard cannot explain extreme precipitation properties. This is implemented assuming the CIs are the effective range (black lines in Fig. 1a) of the national standard, which NOAA suggests as a range of 90% probable precipitation intensity for a specific return period, and the medium of the national standard (red line in Fig. 1a) is a threshold to measure the distance between the national standard and the 21st extreme precipitation.

## Results

### Understanding the background and limitations of the current national standard

**Figure 2:**
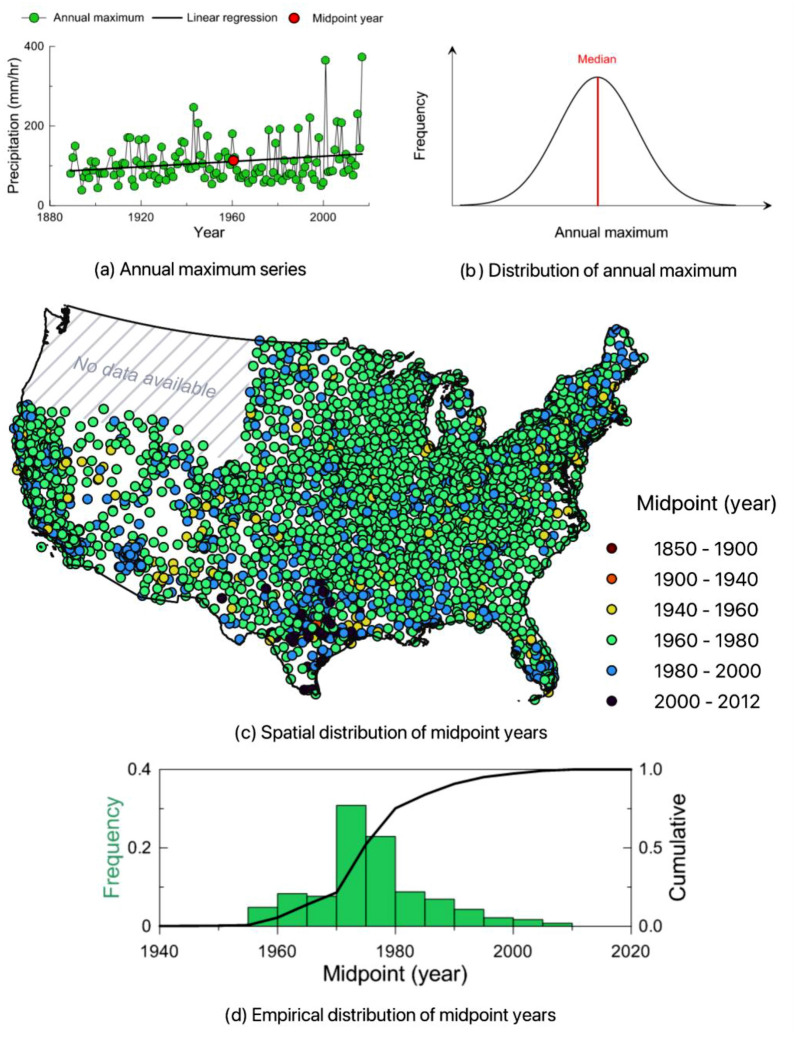
Features of the midpoint of the NOAA Atlas to measure the severity of extreme precipitation. (**a**) Sample of time series of annual maximum precipitation from a station (NOAA Atlas 79-0056 (lat 29.762, lon − 95.358). (**b**) Conceptual distribution of annual maximum precipitation and the location of the median point (midpoint) [the map created by QGIS 3.32.3-Lima (https://qgis.org/en/site/)]. (**c**) Presents the spatial distribution of midpoint years of the NOAA Atlas data. (**d**) Histogram of the midpoint years.

Considering the details of NOAA's regional frequency analysis, the records' median year (hereafter referred to as the midpoint) is a good indicator to understand the centroid of the probability distribution function of the NOAA Atlas 14’s PFEs. This is because the median value of the annual maximum series is strongly correlated with the value at the midpoint. In fact, the index flood, the median value of the annual maximum series as a scale factor in the regional frequency analysis, determines the expectation value of PFEs, and it also represents precipitation depth with a 50% likelihood of occurrence for a given year, corresponding to a 2-year recurrence interval. Figure 2 shows the distribution features of the midpoint of the NOAA Atlas 14 stations. Conceptually, the midpoint stands for the year with the median value of the annual maximum series (Fig. 2b). It could be estimated from a regression line of the annual maximum series (Fig. 2a). In most cases, the midpoint is matched with the year of the medium value, but sometimes it has a difference in a specific location where missing years are identified (because the year data did not pass the quality control test).

The spatial distribution of the midpoints is fairly uniform across the United States, except for the most recently produced volume of the NOAA Atlas 14 (Fig. 2c), which is mostly focused on Texas. Most midpoints (77%) occurred between 1965 and 1985 with the median is 1974 (Fig. 2d). The percentage of the midpoints in the twenty-first century period (2000–2018) is only 3.2%. These statistics suggest that Atlas 14 is most applicable to the time period of 30 years ago, rather than being representative of current conditions in a changing climate, and the impacts associated with its assumption of stationarity should be closely examined. Cumulative percentages of midpoint years are 13.8% (1965 or older), 75.5% (up to 1980), and 91.1% (up to 1990), respectively. In addition, these results suggest that Atlas 14 represents precipitation well into the late twentieth century, after which time it is necessary to include new data and re-consider the assumption of statistical stationarity due to climate change. Collecting all together, this study defines the Atlas 14 as the national standard.

### The severity of the increased extreme precipitation

**Figure 3:**
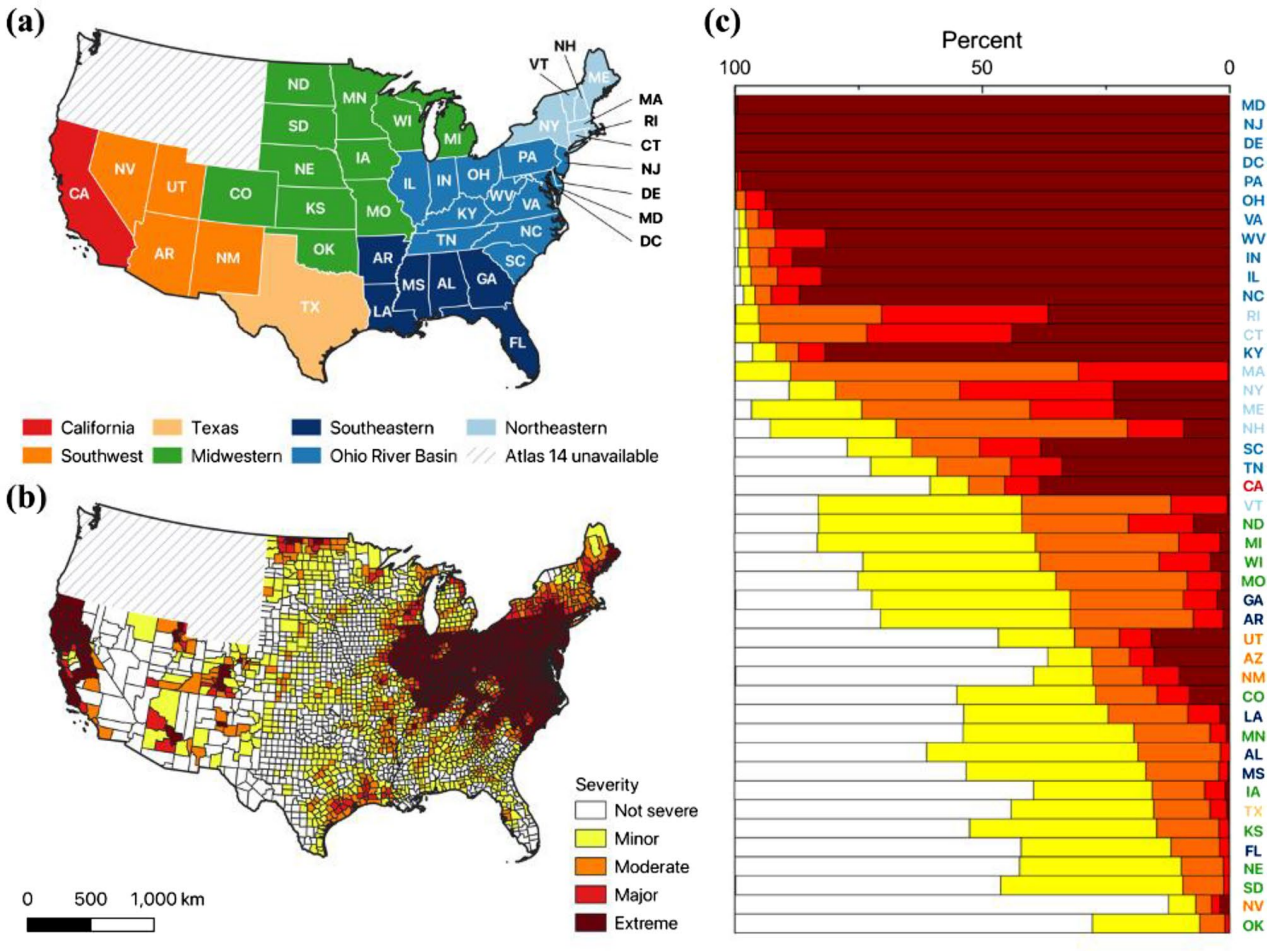
The severity of increased extreme precipitation (a 1-in-100-year event for an hour duration): (**a**) State/region reference map, (**b**) severity map at a county-level scale, and (**c**) the percent area of the four severities by state [the maps created by QGIS 3.32.3-Lima (https://qgis.org/en/site/)].

The spatial and quantitative distributions of the categorized four severity levels by state and county are presented in Fig. 3. 59.9% of the CONUS (excluding WA, OR, ID, MT, WY, where Atlas 14 is not available) are categorized as minor-to-extreme severities, indicating that the 21st-century heavy precipitation has exceeded the current national standard. In other words, the estimated resident population of 259.4 million (79.3% of the U.S. population) in 2,140 counties has experienced or will experience increased heavy precipitation with a higher likelihood than is currently defined.

The counties categorized as the critical severity levels (moderate to extreme) are mostly identified from the Ohio River Basin, Northeastern states, and Northern California, in that order. 80.4% of the Ohio River Basin is categorized as extremely severe, and MD, NJ, DE, and DC are 100% categorized as extremely severe. The primary reasons are increased annual maximum precipitation and its high variability with an upward trend^8,11^. In some regions in CONUS, one of the aforementioned factors has led to increased PFEs from the 21st-century records, but the eastern CONUS areas consistently show that both factors have increased.

The increased extreme precipitation in Northern California is the most notable among the Western states. The percent change in extreme precipitation against the current national standard ranges from 3.6% in Yuba County to 78.4% in Del Norte County. This suggests that most counties in Northern California are experiencing the new normal, and the recent flood events (e.g., multiple precipitation-driven flooding on January 2023) were a part of this change. The occurrence and magnitude of Atmospheric River (AR) events have been suspected as the primary driver of this change^31–34^. Interestingly, the severe areas match where ARs typically make landfall^35–38^.

**Figure 4:**
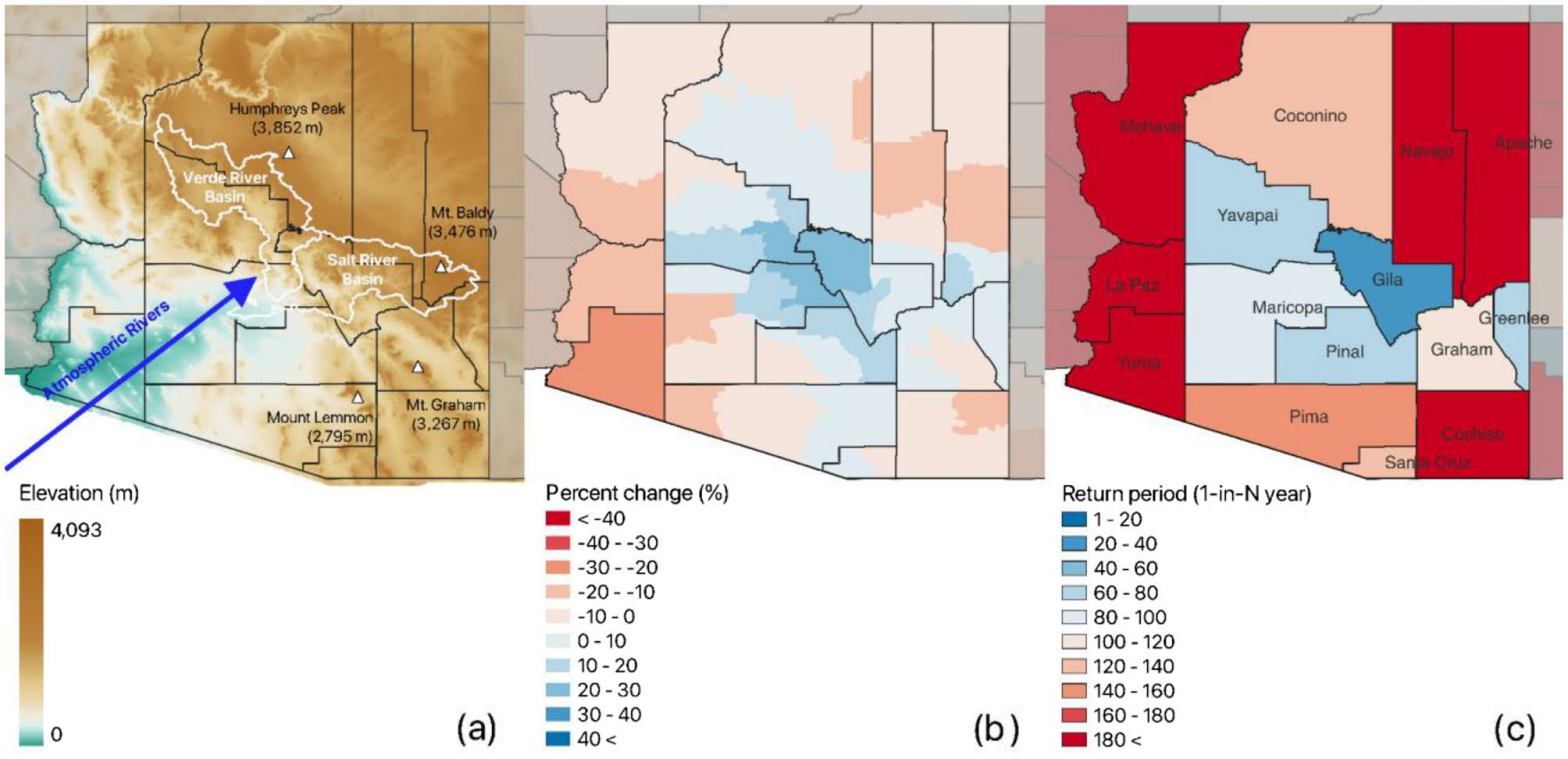
Extreme precipitation changes in Arizona: (**a**) Elevation map, (**b**) percent change in 1-h extreme precipitation, and (**c**) return periods by County [the maps created by QGIS 3.32.3-Lima (https://qgis.org/en/site/)]. The percent change indicates a change of PFEs in the twenty-first century against the NOAA Atlas 14. The percent change is calculated by sub-district in each county. The return period is a converted return period of 1-in-100-year PFEs defined in the NOAA Atlas, based on the new PFEs^8,11^.

Extreme severity is barely identified in most counties in the Midwestern. Still, minor-major severities are spatially confirmed in many areas. Some counties in ND, UT, CO, and AZ present polarized spatial distribution of severities compared to the neighboring counties. This could be explained by their geomorphological features and extreme precipitation sources (e.g., ARs or tropical cyclones). Figure 4 shows geomorphological features and changes in extreme precipitation in AZ as an example. The Verde River and Salt River basins in AZ are where the ARs routinely cross the Baja California Peninsula and influence by pouring heavy precipitation, especially when it crosses the mountain areas due to the orographic effect^41,42^. The counties, Yavapai, Gila, and Pinal, in/near the two basins and within AR’s sphere of influence in general are good examples to describe a link between the impact of AR events and the changes in extreme precipitation^35^. In Gila County, for instance, extreme precipitation for a 1-h duration has increased up to 30%, and a 1-in-100-year storm in the twentieth century now corresponds to a 1-in-39-year storm in the twenty-first century. The severity of the increased extreme precipitation ranges from moderate to extreme. However, considering the return periods ranging from a 1-in-39 to a 1-in-955 year by County, changes in extreme precipitation in AZ are dramatically polarized.

In Texas, the severe areas are characterized near the coastal areas along the Gulf Coast. Tropical cyclones have been found as the primary reason for bringing heavy precipitation into those areas in the early twenty-first century. Their intensity is projected to increase as sea surface temperature increases in the mid-late century, suggesting the increasing trend of extreme precipitation will persist^39^. Increased extreme precipitation in Houston, TX, is notable even though its severity is not classified as extreme. This is because the average annual maximum precipitation in Houston, TX, is the most intense in CONUS, so the increased extreme precipitation greatly impacts the area, even if it does not constitute extreme severity.

We identified not only the increase in extreme precipitation beyond the Atlas 14 national standard but also the decrease in extensive areas of the U.S. 26.9% of counties in CONUS present extreme precipitation likelihood that has decreased against the national standard for a 1-in-100-year return period (equal to or exceeding 1-in-130 year), suggesting that Atlas 14 represents an overestimated design standard for building infrastructures in some U.S. areas. While decreases might result in a safer situation than the increasing extreme precipitation, this also means that new infrastructure based on the current national standard may be over-designed and have excessive costs in those areas.

### The US population at pluvial flood risk

**Figure 5:**
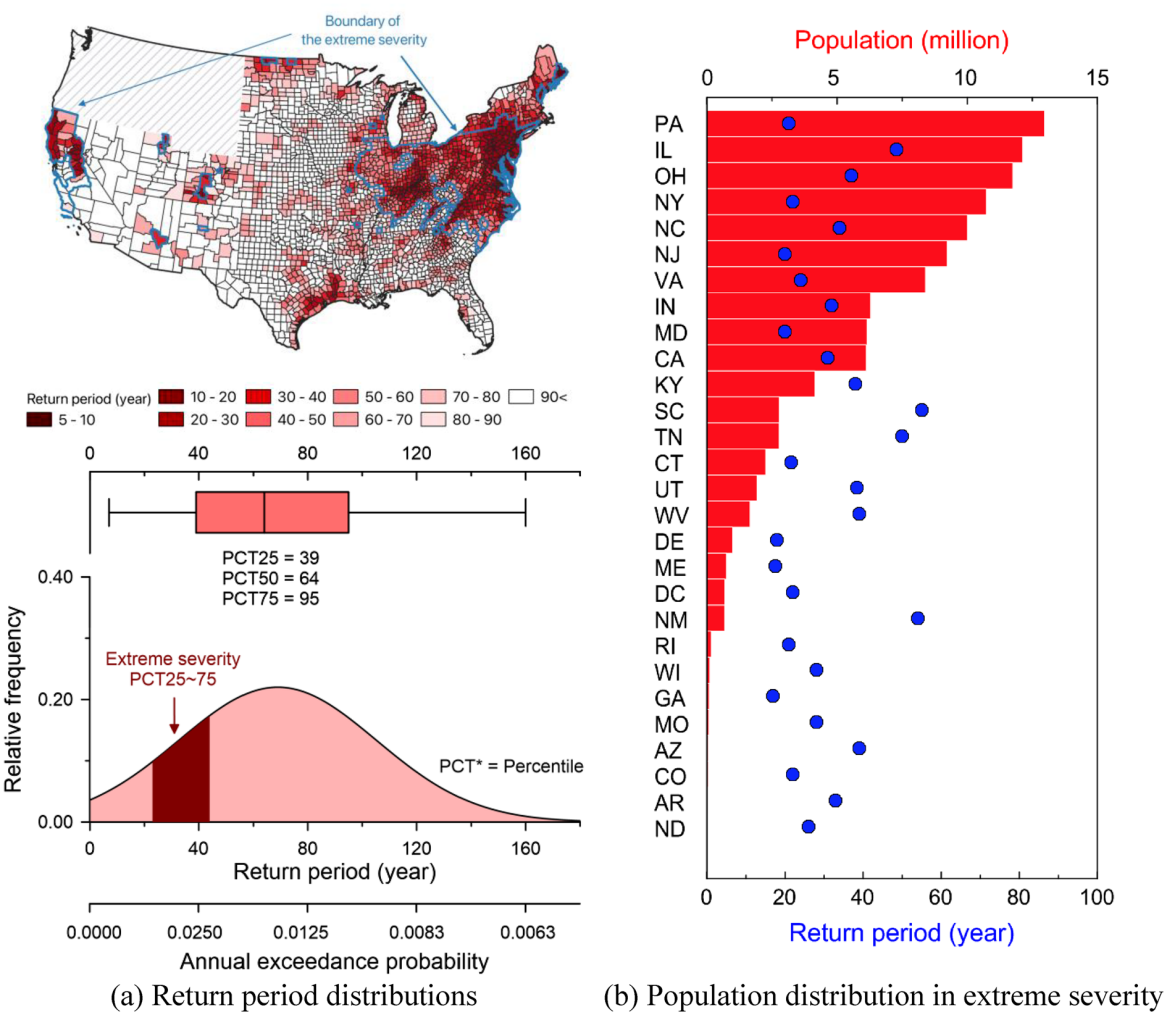
Analysis results of extreme severity counties. Panel (**a**) presents spatial (upper) and quantitative (lower) distributions of median return periods derived from the 21st-century records to precipitation depths corresponding to a 1-in-100-year return period of NOAA Atlas 14 [the map created by QGIS 3.32.3-Lima (https://qgis.org/en/site/)]. The distribution is from the counties categorized as the extreme. Panel (**b**) depicts a distribution of populations exposed to the extreme severity of increased extreme precipitation.

To measure changes in the occurrence likelihood of extreme precipitation, precipitation depths equivalent to a 1-in-100-year return period of the national standard are converted to the twenty-first century’s new return periods. Figure 5 shows (a) the spatial and quantitative distributions of the 21st-century return periods of extreme precipitation and (b) the population distribution exposed to the extreme severity. Overall, the occurrence likelihood of extreme precipitation has increased across the CONUS since the median return period is 64 years. 868 counties classified as extreme severity (29.8% of the total number of counties in the U.S., excluding the Atlas 2 areas showing diagonal lines in Fig. 5a) see the depth associated with their current 1-in-100-year precipitation event occur at least twice as often (less than 50-year) in the same recurrence interval (i.e., occurrence likelihood). This is because their median value of the recurrence intervals of a 1-in-100-year event (a 1% chance of an event occurrence in any given year) is a 1-in-33-year event (a 3% chance of an event occurrence in any given year), which can be interpreted as the factor of the increase is around three times suggesting that three times more likely to occur within a given year.

The results imply that 34.3% of the population, 113 million individuals (the sum of the population bars in Fig. 5b), is currently exposed to severe heavy precipitation risk and associated flood risks. That is, 27.6% of counties are more likely to experience heavy precipitation that could cause stormwater drainage system failure, flooding, property damage, and loss of life. In 39 US counties, the 100-year extreme precipitation event is now a 20-year or lower event, resulting in 5.0% of the total population (over 16.5 million individuals) being five times as likely to experience precipitation levels that are likely to overwhelm much of the infrastructure designed to protect them from flooding. Pennsylvania, Illinois, Ohio, New York, and North Carolina are the top five states at risk for precipitation-driven flooding from the population-at-risk perspective. Texas is not highlighted in the figure due to the polarized spatial pattern of flood risk across the state. However, the median return period of the counties (e.g., Harrison County) near the Gulf of Coast and in the northeast region is 38 years, comparable to the extreme severity counties. Considering that the average annual maximum precipitation for an hour in the areas (e.g., Harris County and its neighboring counties near the Gulf of Mexico) at 166.9 mm/hr is the highest in CONUS, the increased precipitation is also the largest.

## Discussion and conclusions

### Infrastructure at risk due to the changes in extreme precipitation

Across the country, there is a large amount of infrastructure in place where design standards (e.g., 10 ~ 20-year return periods) of less than a 1-in-100-year return period are required. These infrastructure types include highways and urban drainage systems and are projected to frequently and significantly experience precipitation-driven inundation over the coming years. One specific example exists in New Jersey, where the motivation for the project was explicitly driven by the need to protect the roadway against frequent flooding. The designs of this new roadway have been designed to withstand a 10-year national standard, and the stormwater management pipes along the roadway at the lowest point and are designed to a 15-year national standard along the rest of the roadway. According to the results of this study, it will flood every other five years, on average, during its useful life in some regions. The proposed drainage system is intended to mitigate flooding and alleviate deficiencies throughout the project limits. Longitudinal systems and cross-drain pipes are designed for 10-year storm events, while pipes along the roadways that convey runoff from a roadway low point are designed to accommodate a 15-year storm event. Among the improvements being made, (1) the drainage system improvements will reduce ponding water along the roadway gutters, (2) resurfacing and reconstruction of the pavement are needed to provide a service life of 10 to 20 years, and deficiencies in the sidewalks, curb ramps, curbs, guide rail, and traffic signals, along with missing segments of a sidewalk, will also be addressed during construction. To underscore the complexity of the new design, gas mains and connections will be relocated to make space for the new drainage systems, and the outdated water distribution system will be replaced. However, all of this infrastructure and construction is being undertaken using out-of-date precipitation estimates from the NOAA Atlas 14 records, with some adjustments for future observations. By not taking non-stationarity into account, the construction design standards are actually building the roadway to a design standard that more accurately reflects a 1-in-2-year event, which will in turn significantly decrease the useful life of this multi-million roadway construction project.

Various users in science and engineering communities who need a design standard of precipitation for their specific purposes could consider employing new standard values between the upper and middle bounds of Atlas 14 that might capture the non-stationarity of precipitation for the regions where the underestimation of the national standard is highlighted in the previous study^11^. Also, climate adjustment factors based on climate projection data (e.g., CMIP6) could be the other resolution to reflect the recent increases in extreme precipitation and future upward trend. Ultimately, there are a number of approaches that can allow for “business as usual” with a reliable set of precipitation estimates to help ensure resources allocated by the federal, state, and local governments are building infrastructure to a design standard appropriate for protecting local populations and the infrastructure assets themselves.

### Limitations and outlook

The results presented in this study are limited to addressing the changes in extreme precipitation for the given period of time (2002–2021) against the national standard because of the nature of frequency analysis sensitive to historical records. Thus, the recent 2 years (2022–2023) of extreme precipitation storms, including the aforementioned five flooding events at the introduction, were not considered in the analysis, and this data consideration might influence estimating precipitation frequency in the flooding areas and neighboring regions.

This study’s findings demonstrate the need for a new U.S. national standard for extreme precipitation expectations that accounts for the non-stationarity resulting from climate change to secure the safety of life and property. Thankfully, the U.S. Federal government has recognized this need, and NOAA has recently initiated a five-year process to update the standard in two volumes, to be known as NOAA Atlas 15 (https://www.weather.gov/media/owp/hdsc_documents/NOAA_Atlas_15_Flyer.pdf). Volume 1 aims to develop the baseline standard accounting for temporal trends in historical observations with a non-stationarity method. Volume 2 is to develop the future standard by adjusting the baseline standard based on future climate model projections. The new NOAA Atlas 15 will help improve the flood risk assessment and management in the United States and thus enable better infrastructure preparedness and reduce the likelihood of drainage system failure, property damage, and loss of life due to flooding events. Once updated, these adjustments should further allow the Atlas 15 program to indicate non-stationary precipitation changes in the future accurately. However, the standard is expected to be in development for five years (through 2027).

The biggest consequence of this timeline is that there is no agreed-upon guidance to fill the absence of reliable and accurate precipitation estimates between the present and the development of a new national standard, and many planned construction projects will be affected by this lack of an accurate set of estimates to help in the development of appropriate design standards in a changing climate. Additionally, the current precipitation standards for designing transportation infrastructure and urban stormwater drainage systems are often insufficient to protect the public's safety and personal/community property from severe flooding. The flood-prone areas where operating hydraulic and adaptation structures mitigate flood risk urgently need to assess the impact of the increased-hourly extreme precipitation and update their operation rules. Understanding and predicting patterns of heavy precipitation for a short duration would be beneficial in preparing for severe precipitation-driven disasters, such as flash floods and landslides, which are expected to happen more frequently. Following the results of this analysis, the rapid development and dissemination of the next generation of the national standard climatically adjusted to incorporate the new normal according to extreme precipitation projections are strongly recommended.

## Conclusions

Our research addresses how extreme precipitation has severely changed against the national standard and how many populations are affected by this new normal in CONUS. For this purpose, the national standard is evaluated against a newly developed precipitation frequency standard. The notable findings and conclusions can be summarized as follows:

1. The current national standard, NOAA’s Atlas 14, might not be able to offer proper precipitation frequency estimates for all regions in CONUS in the early twenty-first century. Extreme precipitation in almost two-thirds of CONUS has exceeded the national standard, and over three-quarters of the U.S. population is exposed to this type of precipitation-driven flood risk today.
2. In the Ohio River Basin, Northeastern states, and Northern California where the severity of increased extreme precipitation is classified as ‘Extreme’ in this study, the occurrence likelihood of extreme precipitation within a given year has increased by 3–5 times, suggesting that a 1-in-100-year event in the twentieth century is a 1-in-20-year or 1-in-30-year event in the twenty-first century. One-third of the U.S. population is exposed to unprecedented heavy precipitation and associated flood risks, likely overwhelming much of the infrastructure designed to protect them from flooding and resulting in an increased probability of stormwater drainage system failure, flooding, property damage, and loss of life.
3. It is recommended to employ a new climate-adjusted national standard that captures the non-stationarity of precipitation for the regions where the underestimation of the national standard is highlighted in this study.

## Data Availability

The datasets used and/or analyzed during the current study are available from the corresponding author on reasonable request.

## References

[1] Kundzewicz ZW, Kanae S, Seneviratne SI, Handmer J, Nicholls N, Peduzzi P, Mechler R, Bouwer LM, Arnell N, Mach K, Muir-Wood R (2014). Flood risk and climate change: Global and regional perspectives. Hydrol. Sci. J..

[2] Porter K, Wein A, Alpers CN, Baez A, Barnard PL, Carter J, Corsi A, Costner J, Cox D, Das T, Dettinger M (2011). Overview of the ARkStorm scenario. US Geol. Survey Rep..

[3] Keefer TO, Renard KG, Goodrich DC, Heilman P, Unkrich C (2016). Quantifying extreme rainfall events and their hydrologic response in southeastern Arizona. J. Hydrol. Eng..

[4] Markus M, Angel J, Byard G, McConkey S, Zhang C, Cai X, Notaro M, Ashfaq M (2018). Communicating the impacts of projected climate change on heavy rainfall using a weighted ensemble approach. J. Hydrol. Eng..

[5] Lopez-Cantu T, Samaras C (2018). Temporal and spatial evaluation of stormwater engineering standards reveals risks and priorities across the United States. Environ. Res. Lett..

[6] Butcher JB, Zi T, Pickard BR, Job SC, Johnson TE, Groza BA (2021). Efficient statistical approach to develop intensity-duration-frequency curves for precipitation and runoff under future climate. Clim. Change.

[7] Li Z, Tang G, Kirstetter P, Gao S, Li JL, Wen Y, Hong Y (2022). Evaluation of GPM IMERG and its constellations in extreme events over the conterminous United States. J. Hydrol..

[8] Kim J, Shu E, Lai K, Amodeo M, Porter J, Kearns E (2022). Assessment of the standard precipitation frequency estimates in the United States. J. Hydrol. Region. Stud..

[9] Sarhadi A, Ausín MC, Wiper MP, Touma D, Diffenbaugh NS (2018). Multidimensional risk in a nonstationary climate: Joint probability of increasingly severe warm and dry conditions. Sci. Adv..

[10] Slater LJ, Anderson B, Buechel M, Dadson S, Han S, Harrigan S, Kelder T, Kowal K, Lees T, Matthews T, Murphy C (2021). Nonstationary weather and water extremes: A review of methods for their detection, attribution, and management. Hydrol. Earth Syst. Sci..

[11] Kim J, Amodeo M, Kearns E (2023). Atlas of probabilistic extreme precipitation based on the early 21st century records in the United States. J. Hydrol. Region. Stud..

[12] Kunkel KE, Easterling DR, Redmond K, Hubbard K (2003). Temporal variations of extreme precipitation events in the United States: 1895–2000. Geophys. Res. Lett..

[13] Hoerling M, Eischeid J, Perlwitz J, Quan XW, Wolter K, Cheng L (2016). Characterizing recent trends in US heavy precipitation. J. Clim..

[14] Howarth ME, Thorncroft CD, Bosart LF (2019). Changes in extreme precipitation in the northeast United States: 1979–2014. J. Hydrometeorol..

[15] Barbero R, Fowler HJ, Lenderink G, Blenkinsop S (2017). Is the intensification of precipitation extremes with global warming better detected at hourly than daily resolutions?. Geophys. Res. Lett..

[16] Chinita MJ, Richardson M, Teixeira J, Miranda PM (2021). Global mean frequency increases of daily and sub-daily heavy precipitation in ERA5. Environ. Res. Lett..

[17] Utsumi N, Seto S, Kanae S, Maeda EE, Oki T (2011). Does higher surface temperature intensify extreme precipitation?. Geophys. Res. Lett..

[18] Mishra V, Wallace JM, Lettenmaier DP (2012). Relationship between hourly extreme precipitation and local air temperature in the United States. Geophys. Res. Lett..

[19] Zhang X, Zwiers FW, Li G, Wan H, Cannon AJ (2017). Complexity in estimating past and future extreme short-duration rainfall. Nat. Geosci..

[20] Ali, H., Fowler, H. J., Lenderink, G., Lewis, E. & Pritchard, D. Consistent large‐scale response of hourly extreme precipitation to temperature variation over land. *Geophys. Res. Lett.* **48**(4), e2020GL090317 (2021).

[21] Park IH, Min SK (2017). Role of convective precipitation in the relationship between subdaily extreme precipitation and temperature. J. Clim..

[22] Exum NG, Betanzo E, Schwab KJ, Chen TY, Guikema S, Harvey DE (2018). Extreme precipitation, public health emergencies, and safe drinking water in the USA. Curr. Environ. Health Rep..

[23] Kharin VV, Zwiers FW (2000). Changes in the extreme in an ensemble of transient climate simulations with a coupled atmosphere-ocean GCM. J. Clim..

[24] Janssen E, Wuebbles DJ, Kunkel KE, Olsen SC, Goodman A (2014). Observational- and model-based trends and projections of extreme precipitation over the contiguous United States. Earth’s Future.

[25] Soneja S, Jiang C, Upperman CR, Murtugudde R, Mitchell CS, Blythe D, Sapkota AR, Sapkota A (2016). Extreme precipitation events and increased risk of campylobacteriosis in Maryland, USA. Environ. Res..

[26] Prein AF, Rasmussen RM, Ikeda K, Liu C, Clark MP, Holland GJ (2017). The future intensification of hourly precipitation extremes. Nat. Clim. Change.

[27] Akinsanola AA, Kooperman GJ, Pendergrass AG, Hannah WM, Reed KA (2020). Seasonal representation of extreme precipitation indices over the United States in CMIP6 present-day simulations. Environ. Res. Lett..

[28] Lopez‐Cantu, T., Prein, A.F. & Samaras, C. Uncertainties in future US extreme precipitation from downscaled climate projections. *Geophys. Res. Lett.* **47**(9), e2019GL086797 (2020).

[29] Mishra AK, Singh VP (2010). Changes in extreme precipitation in Texas. J. Geophys. Res. Atmos..

[30] Tabari H (2020). Climate change impact on flood and extreme precipitation increases with water availability. Sci. Rep..

[31] Hirota N, Takayabu YN, Kato M, Arakane S (2016). Roles of an atmospheric river and a cutoff low in the extreme precipitation event in Hiroshima on 19 August 2014. Mon. Weather Rev..

[32] Lamjiri MA, Dettinger MD, Ralph FM, Guan B (2017). Hourly storm characteristics along the US West Coast: Role of atmospheric rivers in extreme precipitation. Geophys. Res. Lett..

[33] Zhang W, Villarini G (2018). Uncovering the role of the East Asian jet stream and heterogeneities in atmospheric rivers affecting the western United States. Proc. Natl. Acad. Sci..

[34] Han H, Kim J, Chandrasekar V, Choi J, Lim S (2019). Modeling streamflow enhanced by precipitation from atmospheric river using the NOAA national water model: A case study of the Russian river basin for February 2004. Atmosphere.

[35] Demaria E, Dominguez F, Hu H, von Glinski G, Robles M, Skindlov J, Walter J (2017). Observed hydrologic impacts of landfalling atmospheric rivers in the Salt and Verde river basins of Arizona United States. Water Resour. Res..

[36] Rutz JJ, Steenburgh WJ, Ralph FM (2017). Climatological characteristics of atmospheric rivers and their inland infiltration over the western United States. Mon. Weather Rev..

[37] Cifelli, R., Chandrasekar, V., Chen, H. & Johnson, L.E. High resolution radar quantitative precipitation estimation in the San Francisco Bay area: Rainfall monitoring for the urban environment. *J. Meteorol. Soc. Japan. Ser. II* **96**, 141–155 (2018).

[38] Kim, J., Johnson, L. E., Cifelli, R., Coleman, T., Herdman, L., Martyr-Koller, R., Finzi-Hart, J., Erikson, L. & Barnard, P.L. San Francisco Bay integrated flood forecasting project summary report. *NOAA Technical Memorandum PSD-317* (2018).

[39] Little C, Horton R, Kopp R (2015). Joint projections of US East Coast sea level and storm surge. Nat. Clim. Change.

[40] Huff FA (1967). Time distribution of rainfall in heavy storms. Water Resour. Res..

[41] Hughes M, Mahoney KM, Neiman PJ, Moore BJ, Alexander M, Ralph FM (2014). The landfall and inland penetration of a flood-producing atmospheric river in Arizona: Part II: Sensitivity of modeled precipitation to terrain height and atmospheric river orientation. J. Hydrometeorol..

[42] Rutz JJ, Steenburgh WJ, Ralph FM (2014). Climatological characteristics of atmospheric rivers and their inland penetration over the Western United States. Mon. Weather Rev..

